# Exposure to Indoor Pollutants and Wheeze and Asthma Development during Early Childhood

**DOI:** 10.3390/ijerph120403993

**Published:** 2015-04-13

**Authors:** Evridiki Patelarou, Nikolaos Tzanakis, Frank J. Kelly

**Affiliations:** 1 Florence Nightingale School of Nursing and Midwifery, King’s College London, London SE18WA, UK; 2 Department of Thoracic Medicine, Medical School, University of Crete, Heraklion 71414, Greece; E-Mail: tzanakis@med.uoc.gr; 3 MRC-PHE Centre for Environment and Health, NIHR Environmental Hazards Health Protection Research Unit, King’s College London, London SE19NH, UK; E-Mail: frank.kelly@kcl.ac.uk

**Keywords:** environmental exposures, indoor air pollution, bio-aerosols, respiratory disease, asthma, wheezing, childhood

## Abstract

Background: This review aimed to summarize existing epidemiological evidence of the association between quantitative estimates of indoor air pollution with early childhood respiratory disease. Methods: We carried out a systematic literature search of peer-reviewed epidemiological studies undertaken in “westernized” countries that have assessed exposure to indoor pollutants and asthma and wheeze from infancy up to the age of 5. Results: The search, between January 2004 and February 2014 yielded 1840 studies for consideration. Following application of eligibility criteria to titles and abstracts 22 independent studies were deemed relevant for further review. Two additional studies were next identified through examination of the references’ lists of these studies. Of these 24 selected studies, 16 adopted a prospective cohort design and 8 were case-control studies. Fourteen studies assessed exposure to bio-aerosols, 8 studies assessed exposure to specific air chemicals and two studies assessed exposure to bio-aerosols and air chemicals. Furthermore, 11 studies examined the association of exposure with asthma and 16 with wheeze. Findings indicate that existing studies have reported contradictory effects of indoor pollutants levels and occurrence of asthma/wheeze. Conclusion: Additional research to establish causality and evaluate interventions to prevent disease onset is needed.

## 1. Introduction

Asthma is one of the most common diseases during childhood in Western countries with an increasing prevalence over time and significant morbidity [1]. More than half of all cases of persistent asthma start before the age of 3 and are characterised by bronchial hyperresponsiveness, chronic airway inflammation, recurrent wheezing and mucus- hyper- secretions [2]. Wheezing produced by narrowed airways due to airway inflammation is a frequent symptom for children who are born with small airways and when children have respiratory infections [3,4]. Although epidemiological studies have given an insight into the risk factors that may be associated with an increased risk of asthma, the aetiology of asthma and wheezing remains unclear. Early expression of the asthmatic phenotype represents contributions from a variety of genetic, developmental and immunologic factors [5]. However, there is a growing consensus that these factors do not adequately account for such rapid shift in global prevalence and that early life environmental conditions are of critical importance [6].

Although the relationship between exposure to outdoor air pollutants and health effects has been well documented the effects of indoor pollutants have been studied much less extensively [7]. The World Health Organization estimates that, each year, indoor air pollution (IAP) is responsible for the death of 4.3 million people including young children who are particularly susceptible as their respiratory systems are still forming and their immune systems are not yet mature [7,8,9]. Furthermore people now spend most of their time (90%) indoors which means that the majority of their exposure during a lifetime is air inhaled in the home and therefore most illnesses related to environmental exposures stem from indoor air exposure [10]. Consequently, early life exposure to chemicals and bioaerosolos found at home and their possible roles in allergic airway disease is a critical area of research [11,12,13,14,15].

Allergen exposure is one of the known environmental risk factors associated with the symptoms and the severity of allergy including wheezing and asthma. A systematic review by Chen *et al.* reported contradictory effects of pet exposure on asthma in childhood and resulted in inconsistent recommendations on animal avoidance [16]. Dust mite allergens are also considered as one of the major biogenetic indoor factors supported by strong evidence for a causal relationship with the onset of asthma in susceptible children [17]. Damp homes are also at a higher risk for growing moulds and other damp-dependent bacteria and bio-agents [18]. The most commonly used indicator for bacteria exposure is endotoxin. The association between endotoxin in the environment and allergy and asthma in children has been studied extensively, however, again with inconsistent results [19]. In addition, the extracellular polysaccharide of Penicillium and Aspergillus (EPS-Pen/Asp) is considered a relatively good marker of exposure to indoor moulds, as Penicillium and Aspergillus spp. belong to the most commonly found viable fungi in indoor air. For example, there is preliminary evidence that fungal exposure as estimated by EPS levels in house dust may protect against wheezing [20] and/ or asthma in children [21,22,23]. Finally, exposure to (1-3)-β-d-glucal which can be used as an indicator of early in life fungal exposure and has been associated with a decrease in the risk of physician-diagnosed asthma [24,25]. Notwithstanding this reported evidence, these observations do not provide a conclusive evidence base for a link between such exposures and wheezing/ asthma in young children.

While biological risk factors such as dust mites, bacteria and fungal indicators are known triggers of asthma in some individuals, evidence in support of specific indoor pollutants (nitrogen dioxide, particulate matter, *etc.*) are generally not acknowledged to be significant risk factors for asthma development [26]. The long term exposure to indoor PM and NO_2_ in relation to respiratory symptoms in infants has been studied by several investigators. Although ambiguous, results indicate that efforts to reduce indoor PM and NO_2_ concentrations, especially in inner city homes would likely result in improved pediatric asthma [26,27,28,29,30]. Two recent reviews concluded that exposure to volatile organic compounds (VOCs) consistently increase the risk of asthma like symptoms among children. However, existing studies are acknowledged to be limited by small sample size [31,32]. In a similar manner, a systematic review of the effects of indoor formaldehyde exposure and childhood respiratory health indicated a significant positive association between formaldehyde exposure and childhood asthma [33]. Finally, the Institute of Medicine (IOM) published a report in 2000 which examined how indoor pollutants contribute to asthma and concluded that sufficient evidence of a causal relationship exist between dust mite exposure and asthma onset [34] However, the Committee characterized the evidence of the effect of exposure to other indoor pollutants including other bioaerosols and air chemicals as inadequate-insufficient [34].

In conclusion, previous review articles have considered aspects of the indoor chemical environment but the first effort to bring together the full range of epidemiologic evidence linking indoor chemicals and bioaerosols and childhood asthma and wheezing development was performed 15 years ago. To this extent this review aims to capture and summarize up-to-date epidemiological evidence of the association between quantitative estimates of indoor air pollution with respiratory symptoms during infancy and early childhood including asthma and wheezing.

## 2. Methods

### 2.1. Literature Search Strategy

We performed a systematic search of the existing literature on indoor pollutants and respiratory disease with a focus on asthma-wheezing during infancy and early childhood. The research question which guided this work was: “Given existing epidemiological evidence, what is the relationship between exposure during the first years of life to indoor pollutants and the risk of asthma and wheezing up to the age of 5 years old?” We established a review protocol in advance following standards outlined in the MOOSE Guidelines for Meta-Analyses and Systematic Reviews of Observational Studies [35]. The bibliographic searches used in this review were last updated in February 2014, in PUBMED (National Library of Medicine) and EMBASE.com (Elsevier). Both sources represent the principal international research journals, with each source having its own controlled vocabulary or thesaurus (Mesh in Pubmed and EMTREE in EMBASE.com) to help find the relevant information. The keywords we used and the keywords’ combination are provided in Table 1. The bibliographic search was intended to be exhaustive in scope. To this end, the broadest descriptors were used to identify all articles concerning respiratory disease and IAP. The subject age range was established as 5 years and therefore the limits of “Child, Preschool” and “Infant, Newborn Infant” were used. Full details of the search strategy are presented in Table 1. Bibliographies of each retrieved study and reviews were also checked by hand for additional studies that met broad eligibility criteria.

**Table 1:** Search terms used to identify relevant studies for the review.

IAP and Asthma/Wheezing	
**Exposure**	1. Air Pollution, Indoor/
	2. Particulate Matter/
	3. Nicotine/
	4. Carbon Monoxide/
	5. Nitrogen Dioxide/
	6. Sulfur Dioxide/
	7. Polycyclic Hydrocarbons, Aromatic/
	8. Radon/
	9. Solvents/
	10. Asbestos/
	11. Ozone/
	12. Pesticides/
	13. Volatile Organic Compounds/
	14. Formaldehyde/
	15. Benzene/
	16. Toluene/
	17. Styrene/
	18. Dibutyl Phthalate/ or phthalate.mp.
	19. Polyvinyl Chloride/
	20. Allergens/
	21. Mites/
	22. Cockroaches/
	23. Endotoxins/
**24. 1 or 2 or 3 or 4 or 5 or 6 or 7 or 8 or 9 or 10 or 11 or 12 or 13 or 14 or 15 or 16 or 17 or 18 or 19 or 20 or 21 or 22 or 23**	
**Outcome**	Asthma/ Respiratory Sounds/ Wheezing
**25. 1 or 2 or 3**	
**26. 24 and 25**	

Abbreviation: IAP, indoor air pollution.

### 2.2. Selection Criteria

We focused on studies that recorded at least one indicator of respiratory health and which included a measured estimation of indoor exposure in relation to the respiratory health of children under the age of 5 years old. We restricted our search to preschool years as environmental exposures during this age are mainly related to indoor sources of exposures as children of this age spend about 90% of their time indoors (mainly home environment). Furthermore, we were particularly interested in this age group as the preschool children have higher asthma morbidity than do any other age group and therefore our review could inform the development of future interventional studies to prevent asthma onset in early life. Specific inclusion and exclusion criteria were set up and guided out review.

The inclusion criteria for this systematic review were:

- papers published in peer-reviewed journal
- papers published in English language
- papers published during the last 10 years as up-to-date knowledge was considered necessary
- human epidemiological observational studies of any study design
- studies evaluating IAP at home
- studies that performed quantitative estimates of exposure

The exclusion criteria for this systematic review included:

- studies conducted in developing countries
- studies examining respiratory outcomes other than asthma or wheezing
- studies examining asthma/wheezing after the age of 5
- studies that did not include a measure of effect for at least one outcome associated with the indoor exposure of interest
- interventional studies

We did not include studies undertaken in developing countries, because some of the indoor factors such as burning of biomass fuel in open fireplaces indoors are very specific and are not common in “westernized” countries. Studies not meeting these criteria were excluded and studies meeting the criteria were shortlisted for inclusion in the review.

### 2.3. Literature Screening and Data Extraction

Studies were evaluated for inclusion by two independent reviewers for relevance to the subject. Study selection was accomplished through four levels of study screening. Disagreement was resolved by discussion. At level 1 screening, studies were excluded by reviewing the title of the article. At level 2 screening, abstracts of all studies accepted at level 1 were reviewed for relevance. For level 3 screening, the full text was obtained for relevant papers and any citations for which a decision could not be made from the abstract. For level 4 screening, a hand search of recent reviews or already retrieved original articles was performed and additional referenced, manuscripts were included in the systematic review. Information on study design, methods, pollutants and outcome of interest, source and timing of exposure, location of study, results and confounding factors were obtained by using a previously piloted data extraction form.

## 3. Results

### 3.1. Bibliographic Search

**Figure 1:**
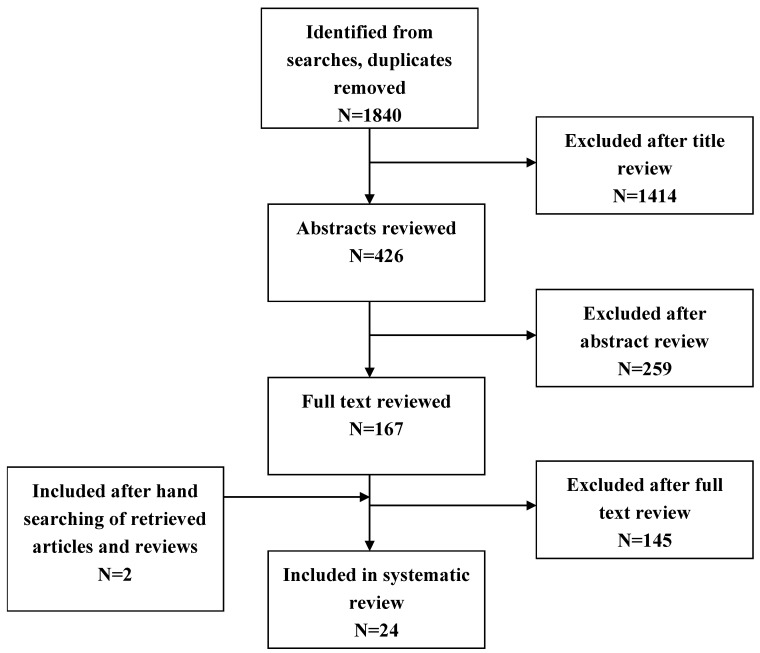
Flow chart for selection of studies.

Our combined search to MEDLINE and EMBASE retrieved 1840 records. The initial screening of manuscript titles and abstracts excluded 1680 records. We excluded another 138 articles after examination of the full text as they did not meet the selection criteria. Additionally, searching the reference lists of retrieved reviews and articles identified two further articles. Figure 1 shows the numbers of studies identified and selected/excluded in each phase of the search. Ultimately, 24 articles were deemed suitable for inclusion in the review.

### 3.2. Overview of the Included Studies

Characteristics of the studies included in the analysis are given in Table 2. Among the relevant articles, 16 referred to prospective cohort studies [36,37,38,39,40,41,42,43,44,45,46,47,48,49,50,51] and eight to case-control studies [52,53,54,55,56,57,58,59]. Five prospective cohort studies had, as a population, healthy children at risk of developing the disease (wheezing or asthma) defined either as “at least other sibling with the disease” or “maternal or paternal history of the specific disease or atopy” [37,38,42,49,50]. Ten studies were conducted in US [37,41,42,43,44,48,50,51,55,58], three in Sweden [52,53,56], two in Netherlands [36,38], two in Finland [47,54], two in Poland [45,46], one in Australia [57], one in Germany [40], one in UK [59], one in Denmark [49] and one in New Zealand [39].

**Table 2:** An overview of studies’ characteristics, exposure and outcome definitions.

Author *et al.* (Year) [Ref.]	Study Characteristics	Exposure		Outcome	
	Setting, Participants, Study Duration and Design	Pollutants of Interest	Exposure Assessment (Sample, Home Area)	Outcome of Interest	Definition
Bornehag *et al.* (2004) [11]	Sweden, Värmland, 2001–2002; Nested case-control study; 198 symptomatic and 202 controls; 3–8 years old	Phthalates	Dust samples Children’s bedroom	Asthma	Physicians’ diagnosis Questionnaire
Rumchev *et al.* (2004) [57]	Australia, Perth, 1997–1999; Case-control study; 88 asthmatic and104 controls	VOCs	Air-Dust samples; Living room	Asthma	Questionnaire Physicians’ diagnosis
van Strien *et al.* (2004) [51]	US, Connecticut and Massachusetts,1996–1998; Cohort study; 768 infants	NO2 Nitrous acid	Air samples; Living room	Wheezing	Maternal report
Brussee *et al.* (2005) [36]	Netherlands, Utrecht, 1995–1997; Prenatal clinics; 1127 children; (atopic and non-atopic mothers); First 4 years of life	Dust mites Cat allergen Dog allergen	Dust samples; Children’s bedroom	Wheezing Asthma	Questionnaire Physicians’ diagnosis
Heudorf *et al.* (2005) [40]	Germany, Frankfurt, 1998; 287 children; Under 6 years old	Parquet glue Benzo(a)pyrene Dust mites	Air sample	Wheezing	ISAAC questionnaire (modified)
Tavernier *et al.* (2005) [59]	UK, Manchester, 1999; Case-control study (IPEADAM study); 105 asthmatic and 95 controls; 4 to 17 years old	Dampness Endotoxin Dust-mite PM2.5 VOCs NO2 Formaldehyde	Air-dust sample; Living room—Children’s bedroom	Asthma	Questionnaire Physicians’ diagnosis
Campo *et al.* (2006) [37]	Ohio, US; Cohort study; Childhood Asthma and Air Pollution study; 532 infants born to atopic parents	Endotoxin	Dust samples; Infant’s primary activity room	Wheezing	Questionnaire Physicians’ diagnosis
Douwes *et al.* (2006) [38]	Netherlands, Utrecht, 1996–1997; Cohort study (PIAMA study); 696 subjects of atopic mothers; 4 years old of age	Endotoxin EPS-*Pen/Asp* (1→3)-β-d-glucans	Dust sample; Children’s bedroom	Asthma Wheezing	Questionnaire
Gillespie *et al.* (2006) [39]	New Zealand, 2001; Cohort study; 881 infants	Endotoxin	Dust samples; Children’s bedroom	Wheezing	Questionnaire
Horick *et al.* (2006) [41]	US, Boston, 1994–1996; Cohort study 404 infants	Endotoxin	Dust sample; Living room	Wheezing	Questionnaire
Hyvärinen *et al.* (2006) [54]	Finland, Kuopio; Case-control study; Kuopio University Hospital; 36 asthmatic children; 36 non-asthmatic children; 12–84 months of age	Fungal biomass/ergosterol 3-OH fatty acids LPS Viable bacteria Mesophilic actinomycetes Viable fungi	Dust sample	Asthma	Admission to the hospital
Perzanowski *et al.* (2006) [48]	US, New York City; Cohort study; 301 children; 3 years old	Endotoxin	Dust sample; Children’s bedroom	Wheezing	Questionnaire
Surdu *et al.* (2006) [58]	US, Canada; Case-control study; 25 asthmatic and 25 controls; 2–14 years old	Dust mites Cat allergen	Air-dust sample; Living room—Children’s bedroom	Asthma	Questionnaire
Iossifova *et al.* (2007) [43]	US, Ohio, 2001–2003; Cohort study, 574 infants	(1–3)-β-d-glucan Endotoxin	Dust samples; Baby’s primary activity room	Recurrent wheezing	Questionnaire
Jedrychowski *et al.* (2007) [46]	Poland, Krakow, 2005; Cohort study; 275 children; 3 years old	Dust mites	Dust samples; Children’s bedrooms—Kitchen floors	Wheezing Number of wheezing episodes and their duration.	Questionnaire
Iossifova *et al.* (2009) [44]	US, Ohio, 2001–2003; Prospective cohort study; 483 children at the age of 3 years old	(1–3)-β-d-glucan Endotoxin	Dust samples; Child’s primary activity room	Asthma risk	Maternal report
Raaschou-Nielsen *et al.* (2010) [49]	Copenhagen, Denmark; 411 infants of mothers with asthma	NOx NO2 PM2.5 Black carbon Formaldehyde	Air sample; Children’s bedroom	Wheezing	Parental report
Choi *et al.* (2010) [53]	Sweden, 2001–2002; Case-control study; 198 asthmatic and 202 controls; 3–8 years of age	VOCs	Air-dust samples	Asthma	Questionnaire Physicians’ diagnosis
Rosenbaum *et al.* (2010) [50]	US, New York 2001–2002; Cohort study; 103 infants at risk for asthma	PM Combustion gases VOCs Viable fungi Bacteria Allergen Endotoxin	Air-dust samples; Living room	Doctor diagnosed asthma	Nurse practitioner’s diagnosis Questionnaire
Hunt *et al.* (2011) [42]	US, New York; Birth cohort whose mothers had a diagnosis of asthma	PM	Air samples	Wheezing	Parental report Nurse practitioner’s diagnosis
Jones *et al.* (2011) [55]	US, New York; Case-control study; 50 asthmatic and 49 controls; 3 to 17 years old	Fungal flora	Air samples; Living room—Family room	Asthma	Questionnaire
Karvonen *et al.* (2012) [47]	Austria, Finland, France, Germany, Switzerland; Prospective cohort study; 1133 children; 2 years old	Endotoxin EPS-*Pen/Asp*	Dust samples; Living room—Mother’s mattress	Asthma Wheezing	Questionnaire based. Parental report Physicians’ diagnosis
Moniruzzaman *et al.* (2012) [56]	Karlstad, Sweden, 2001–2002; Case-control study; 198 asthmatic and 202 controls; 1 to 6 years old	Dust samples	Dust samples; Living room—Children’s bedroom	Asthma	Questionnaire Physicians’ diagnosis
Jedrychowski *et al.* (2014) [45]	Poland, Krakow; Prospective cohort study; 257 children; 4 years old	PAH	Air samples	Wheezing	Questionnaire

Overall, among the retrieved studies exposure characterisation varied widely, particularly in terms of the exposure of interest and exposure assessment methodology. In total, 14 studies examined indoor exposure to bioaerosols [36,37,38,39,41,43,44,46,47,48,54,55,56,58], eight to air chemicals [40,42,45,49,51,52,53,57] and two assessed both bio-aerosols and specific chemicals [50,59]. In terms of the exposure to bioaerosols, 11 studies investigated exposure to endotoxin [37,38,39,41,43,44,47,48,50,56,59], three articles exposure to dust mites [36,46,59], three to fungi flora [50,54,55], three to glycans [38,43,44], two to EPS-Pen/Asp [38,47], two to LPS [50,54], one to pet allergens [36], one to fungal biomass ergosterol and one to bacteria fatty acids [54]. In addition, three studies assessed exposure to NO_2_ [49,51,59], three to PM [42,49,59], three to VOCs [53,57,59], two to formaldehyde [49,59], one to phthalates [52] and one to PAHs [40].

Dust samples were used for bioaerosolos’ assessment in 18 studies [36,37,38,39,40,41,43,44,46,47,48,50,53,54,56,57,58,59]. Air samples were also used for air chemicals’ analysis (NO_2_, PM, phthalates, VOCs, black carbon, nitrous acid, NO_x_) by 12 studies [40,42,45,49,50,51,52,53,55,57,58,59]. All studies performed measurements in one or more of the main rooms of the house where children spent most of their time including the living room, the bedroom and the kitchen. None of the studies took into account indoor exposure in places other than home, including child-care settings. Dust samples were collected with the use of vacuum cleaners mainly from surfaces including floors, carpets, rugs, mattresses and shelves and air samples were mainly collected with the use of passive air samples (Palmes tubes).

In terms of the outcome of interest asthma was the primary outcome for 10 studies [36,38,52,53,54,55,56,57,58,59], risk of asthma for one study [44] and wheezing for 16 studies [36,37,38,39,40,41,42,43,44,45,46,47,48,49,50,51]. Asthma was assessed with the use of questionnaires by two studies [55,58], was based on doctor-diagnosis for four studies [36,38,47,54] and, finally, four studies validated doctor’s diagnosis by questionnaires [53,56,57,59]. Similarly, wheezing definition was assessed through questionnaires by 12 studies [36,38,39,40,41,43,44,45,46,48,49,51], was based on doctor-diagnosis for two studies [42,47] and two studies validated doctor-diagnosis by questionnaires [37,50].

### 3.3. Effects of Quantified Exposure to Indoor Pollutants on Asthma and Wheezing

#### 3.3.1. Sufficient Evidence of Endotoxin Exposure and Outcome Association

Endotoxin was the exposure of interest for 11 studies and the only exposure metric for which evidence is sufficient for an exposure-outcome (wheezing and asthma) association.

Six studies investigated the effect of endotoxin exposure on wheezing, all published between 2005 and 2012 [37,39,41,43,48,50]. Four of these six studies found a positive association between endotoxin exposure and wheezing [39,41,48,50], one found an inverse association [37] and one reported no association [43]. All studies were prospective cohorts [37,39,41,43,48,50], used similar exposure assessment methods (dust samples collected either from child’s bedroom, main activity or living room) and assessed outcome mainly with the use of questionnaires. Sample size and children’s age varied significantly among studies. However, the most recent study and the largest cohort both confirmed an increased risk of wheezing among children exposed to higher levels of endotoxin [39,50]. A short overview of each study in chronological order of publication (from oldest to most recent) is presented below.

Campo *et al.* published the first cohort study that evaluated the relationship among high endotoxin exposure and wheezing [37]. This study was conducted among high-risk infants and showed that the risk for recurrent wheezing (aOR 0.4 95%CI 0.1–0.9) and any wheezing (aOR 0.3 95%CI 0.1–0.8) was significantly less in homes with high endotoxin exposure in the presence of 2 or more dogs [37]. In contrast, in the same year another cohort study reported that wheezing at 15 months was significantly associated with higher endotoxin levels (OR 1.54 95%CI 1.03–2.30) particularly among infants with a parental history of allergic disease (OR 1.67 95%CI 1.07–2.60) [39]. Similarly, Horick *et al.* examined the relationship between endotoxin exposure and wheezing and found that endotoxin was significantly associated with a nearly 6-fold increase in the prevalence of wheezing for a one interquartile range increase in airborne endotoxin (95%CI 1.2–26) [41]. In line with the above, Perzanowski *et al.* showed that children in homes with higher endotoxin concentration were more likely to wheeze at the age of 2 years (OR 1.34 95%CI 1.01–1.78) and that these associations were stronger among children with a maternal history of asthma [48]. On the other hand, Iossifova *et al.* measured endotoxin levels in settled dust collected from infants’ primary activity rooms and found that exposure to endotoxin had no effect on recurrent wheezing [43]. However, later findings by Rosenbaum *et al.* who evaluated the associations between exposure to endotoxin and episodes of wheezing in a cohort of 103 infants at risk for asthma reported that high indoor levels of endotoxin (<100 *vs.* ≥100 EU/mg) were a significant risk factor for wheezing (OR 2.62 95%CI 1.12–6.13) in the first year of life [50].

Five studies investigated endotoxin exposure and asthma as the main outcome of interest [38,44,47,56,59]. Two of these studies reported an inverse association of endotoxin exposure and asthma [38,47], two found a positive association [56,59] and one reported no association [44]. All five studies were very similar regarding the exposure and outcome assessment methodology adopted. However, a prospective cohort design with adequate number of participants was adopted by two studies [38,47]. The first cohort of children with atopic mothers found that floor levels of endotoxin were inversely associated with asthma (OR 0.40 95%CI 0.21–0.77) [38] and the most recently published cohort of 1133 children confirmed an inverse association between incidence of asthma and levels of endotoxin assessed in the mother’s mattress (aOR 0.79 95%CI 0.60–1.05) and in living room (aOR 0.71 95%CI 0.51–0.99) [47]. The two studies that found a positive association of endotoxin exposure and asthma were case-control studies with particularly small number of cases and controls, and therefore their results need to be interpreted with caution [56,59]. Specifically, Tavernier *et al.* published in 2005 the first case-control study that investigated exposure to endotoxin in a population of asthmatic and healthy children and found that in the living room carpets the levels of endotoxin were significantly higher in the homes of asthmatic children compared with their non-asthmatic control (OR 1.88 95%CI 1.11–3.18) [59]. Similarly, findings of another case-control study showed an increased risk for asthma for the upper quartile of exposure to endotoxin in the child’s bedroom (aOR 2.31, *p*-value 0.016) [56].

#### 3.3.2. Suggestive Evidence of Fungal Indicators (EPS-Pen/Asp and (1->3)-β-d-glucans) and Outcome Association

Summary evidence of exposure to EPS-Pen/Asp and (1->3)-β-d-glucans is suggestive. Two studies assessed exposure to fungal EPS-Pen/Asp [38,47] and two to (1->3)-β-d-glucans [38,43]. Fungal EPS-Pen/Asp exposure was associated with decreased risk for asthma and this finding was confirmed by two prospective cohort studies with very similar exposure and outcome assessment methodologies [38,47]. On the other hand, no additional studies have been published since 2012 to confirm or challenge this finding and therefore further research is required. Below we present a short description of each study.

Douwes *et al.* assessed the association between microbial exposure at 3 months and the development of doctor-diagnosed asthma and wheezing in the first 4 years in a birth cohort study of children with atopic mothers [38]. Fungal (1->3)-β-d-glucans and EPS-Pen/Asp and dust on living room floors were measured at 3 months of age. Study showed that higher floor levels of EPS-Pen/Asp were inversely associated with doctor diagnosed asthma (OR 0.42 95%CI 0.18–0.99) and persistent wheezing (OR 0.37 95% CI 0.15–0.96). Higher levels of exposure to fungal (1->3)-β-d-glucans were not found to have a statistical significant association with either asthma (aOR 0.70 95%CI 0.30–1.60) or wheezing (aOR 0.37 95%CI 0.15–0.96) [38]. The association between (1–3)-β-d-glucan exposure and wheezing/risk of asthma up to the age of five years old was also studied by Iossifova *et al.* [43,44]. (1–3)-β-d-glucan was measured in settled dust collected from infants’ (born to atopic parents) primary activity rooms and higher levels of exposure were strongly associated with reduced likelihood of recurrent wheezing (aOR 0.39 95%CI 0.16–0.93) [43]. The same study showed that children aged 3 years exposed to low concentration of (1–3)-β-d-glucan levels (≥22 μg/g) were at increased risk of a positive API (aOR 3.4 95%CI 0.5–23.5), whereas those with high (1–3)-β-d-glucan levels (>133 μg/g) were at decreased risk of a positive API (aOR 0.6 95%CI 0.2–1.6) but the associations were not statistically significant [44]. Finally, Karvonen *et al.* in a birth cohort determined EPS-Pen/Asp from living room floor and mother’s mattress dust samples collected at 2 months of age and showed that the incidence of asthma was inversely associated with the loads (units/m2) of EPS-Pen/Asp (aOR 0.75 95%CI 0.55–1.04) [47].

#### 3.3.3. Insufficient Evidence of other Fungal and Bacteria Indicators and Outcome Association

Mould and fungal concentrations were also investigated by three studies but findings are inconclusive [50,54,55]. Hyvärinen *et al.* studied the determinants of ergosterol, 3-OH fatty acids and viable microbes in vacuum cleaner dust and investigated the association between these microbial markers and childhood asthma [54]. These authors reported that higher levels of actinomycetes, ergosterol and viable fungi tended to increase the risk of asthma although the associations did not quite reach statistical significance [54]. Later Rosenbaum *et al.* assessed exposure to airborne fungal levels and episodes of wheezing in a cohort of 103 infants at risk for asthma (due to maternal history of asthma) living primarily in low-income urban settings [50]. Analysis showed that high indoor levels of Penicillium were a significant risk factor for wheezing (OR 6.18 95%CI 1.34–28.46) in the first year of life [50]. Furthermore, Jones *et al.* examined the relationship between airborne mould concentrations and asthma status among children and aimed to identify the contribution from specific mould genera in air [55]. However, no significant differences in mean fungal concentrations between the homes of cases and controls were observed, although the rate of exposure to several moulds was higher among the cases [55].

#### 3.3.4. Insufficient Evidence of Dust and Pet Allergens and Outcome Association

Three studies were published between 2005 and 2007 and examined the effect of indoor dust and/or pet allergen exposure including exposure to dust mites and pet allergens [36,46,59]. One study explored the association between pets’ allergen exposure in the indoor environment but investigators found no significant associations between cat and dog allergen exposure and persistent wheezing [36]. Dust mite exposure and wheezing evidence of association is also insufficient as only one study has shown a strong positive association with duration and episodes of wheezing (aIRR 1.84 95%CI 1.45–2.34 and aIRR 1.56 95%CI 0.88–2.75, respectively) [46] and others reported no association [36,59]. However, findings are based on a small cohort study (275 participants) and should be interpreted with caution. Future well designed epidemiological studies need to explore the role of dust and pet allergens on children’s respiratory health.

#### 3.3.5. Insufficient Evidence of Indoor Air Chemicals and Outcome Association

Four studies in total quantified indoor chemical exposure including nitrogen dioxide (NO_2_), VOCs and particulate matter (PM) [49,51,57,59]. However, findings are insufficient to conclude of an exposure and outcome association as the primary pollutant-outcome of interest and study designs differ significantly between studies. Other reported chemical pollutants including formaldehyde, PAH, phthalates, black carbon have been rarely examined [40,49,52]. Below we summarise the main findings of each study by pollutant of interest.

vanStrien *et al.* studied 768 infants who were at risk for developing asthma and measured NO_2_ and NO concentrations on a single occasion in the homes of infants [51]. These authors concluded that infants living in homes with an NO_2_ concentration exceeding 17.4 ppb (highest quartile) had a higher frequency of days with wheezing (RR 2.2 95%CI 1.4–3.4) when compared with infants in homes that had NO_2_ concentrations lower than 5.1 ppb (lowest quartile) after controlling for nitrous acid concentration [51]. Furthermore, Tavernier *et al.* in their case-control study found that higher levels of NO_2_ in living room and bedroom were not associated with an increased risk of asthma [59]. Another cohort study of 411 infants assessed the impact of measured long-term exposure to indoor air pollution including NO_2_ and NOx on wheezing symptoms in infants and found no systematic association between risk for wheezing symptoms and the levels of these air pollutants [49].

Rumchev *et al.* in their case-control study investigated the association between domestic exposure to VOCs and asthma in young children [57]. Most of the individual VOCs appeared to be significant risk factors for asthma with the highest ORs for benzene followed by ethylbenzene and toluene (Table 3). Another case-control study showed that there were not significant different levels of benzene exposure in the homes (bedrooms measurements) of cases compared to those of controls (OR 0.59 95%CI 0.26–1.31) [59]. Choi *et al.* also examined the residential concentrations of VOCs in pre-school age children in Sweden in a case-control investigation and showed that a natural-log unit of summed propylene glycol and glycol ethers in bedroom air (equal to interquartile range: 3.43–15.65 mg/m^3^) was associated with 1.5 fold greater likelihood of asthma (95%CI 1.0–2.3) [52].

**Table 3:** An overview of studies’ statistically significant findings.

Author (Year)	Main Results	Confounders
Bornehag *et al.* (2004) [11]	Asthma; DEHP 0.00–0.46 *vs.* 0.46–0.77 *vs.* 0.77–1.30 *vs.* 1.30–40.46mg/g; Referent *vs.* aOR 1.56 (0.70***–***3.46) *vs.* aOR 2.05 (0.94***–***4.47) *vs.* aOR 2.93 (1.36***–***6.34)	Sex, age, smoking at home, type of building, construction period, self-reported, flooding during preceding 3 years, the other phthalate variable
Rumchev *et al.* (2004) [57]	**Asthma; *Total VOC (with 1 μg/m^3^ increase in VOC exposure)*** aOR 1.02 95%CI (1.02–1.03) ***Total VOC (with 10 μg/m^3^ increase in VOC exposure)*** aOR 1.27 95%CI (1.18–1.37) ; ***Benzene*** aOR 2.92 95%CI (2.25–3.80) ***Toluene*** aOR 1.84 95%CI (1.41–2.41) ***Ethylbenzene*** aOR 2.54 95%CI (1.16–5.57) ***Mxylene*** aOR 1.61 95%CI (1.10–2.35) ***Dichlora*** aOR 1.55 95%CI (1.27–1.89) ***Tolichlar*** aOR 1.30 95%CI (1.07–1.58)	Age, sex, atopy, socioeconomic status, smoking indoors, presence of air conditioning and house dust mites
van Strien *et al.* (2004) [51]	No statistically significant associations reported.	Season of sampling, parental asthma diagnosis, mother’s ethnic background, mother’s educational level, smoking in the home, day care, living in an apartment, the presence of siblings, sex, other contaminant
Brussee *et al.* (2005) [36]	No statistically significant associations reported.	Atopy of the parents, sex, study region, education of the mother, presence of older siblings, contact with children other than siblings, and exposure to environmental tobacco smoke
Heudorf *et al.* (2005) [40]	No statistically significant associations reported.	
Tavernier *et al.* (2005) [59]	No statistically significant associations reported.	
Campo *et al.* (2006) [37]	**Recurrent wheezing; *Endotoxin (EU mg)by the number of dogs in the home (≥2)*** Referent *vs.* OR 0.4 95%CI (0.1–0.9) **Any wheezing; *Endotoxin (EU/mg) by the number of dogs in the home (≥2)*** Referent *vs.* OR 0.3 95%CI (0.1–0.8)	
Douwes *et al.* (2006) [38]	**Asthma; *Endotoxin (EU/m^2^) ≤ 142.2 vs. 142.3−<1657.2 vs. ≥1657.2*** Referent *vs.* OR 0.47 95%CI (0.26–0.86) *vs.* OR 0.40 95%CI (0.21–0.77) ***EPS (EPSU/m^2^) < 365.1 vs. 365.1−<8802.3 vs. ≥8802.3*** Referent *vs.* OR 0.78 95%CI (0.40–1.55) *vs.* OR 0.42 95%CI (0.18–0.99) **Wheezing persistent in past 4 years *EPS (EPSU/m^2^) < 365.1 vs. 365.1–<8802.3 vs. ≥8802.3*** Referent *vs.* OR 1.07 95%CI (0.53–2.16) *vs.* OR 0.37 95%CI (0.15–0.96).	Sex, region, parental education level, exposure to indoor tobacco smoke in the past 4 years, other children in the household at 4 years of age
Gillespie *et al.* (2006) [39]	**Wheezing; *Endotoxin (EU/g) < 4621 vs. 4621–10.749 vs. 10750–23672 vs. >23672 *** Referent *vs.* OR 1.08 95%CI (0.72–1.62) *vs.* OR 1.23 95%CI (0.82–1.84) *vs.* OR 1.54 95%CI (1.03–2.30)	Total number of people in the house, total number of rooms in the house, owning a pet, having a damp, musty smell, dampness or mold in the bedroom, having an open fireplace, maternal smoking, type of flooring in the bedroom, NewZealand deprivation index
Horick *et al.* (2006) [41]	**Wheezing; *Endotoxin (EU/g)—Estimated RR reflects an increase of one interquartile range (0.34 log_10_) in dust endotoxin exposure*** aRR 1.45 95%CI (1.20–1.76) aRR 5.56 95%CI (1.19–26.03) corrected for measurement error	
Hyvärinen *et al.* (2006) [54]	No statistically significant associations found.	Parental asthma, father’s education, number of siblings, having livestock, having moisture damage in living quarters, daycare attendance
Perzanowski *et al.* (2006) [48]	**Wheezing 13−24 months; *GM of endotoxin concentration 75.9EU/mg*** OR 1.3 95%CI (1.01–1.78)	
Surdu *et al.* (2006) [58]	**Asthma; *Fel d 1 < 8 vs ≥8 μg/g dust*** OR 3.45 (PAR) (9.3%) ***<1 vs. ≥1 μg/g dust*** OR 1.06 (PAR) (1.8%)	None
Iossifova *et al.* (2007) [44]	**Recurrent wheezing; *(1–3)-β-d-glucan <3 vs. 3-22 vs. 23-60 vs. 61–133 vs. 134–900 (μg/g)*** aOR 3.04 95%CI (1.25–7.38) *vs.* aOR 1.29 95%CI (0.99–1.67) *vs.* aOR 0.82 95%CI (0.65–1.05) *vs.* aOR 0.39 95%CI (0.16–0.93)	Day-care attendance, either parent asthma, gender, race, number of siblings in the same household, visible mold in home, mother’s smoking, lower respiratory condition, and upper respiratory condition
Jedrychowski *et al.* (2007) [46]	**Wheezing days; *HDM level ≤2 vs. >2μg/g*** aIRR 1.84 95%CI (1.45–2.34)	House dampness, indoor moulds, maternal allergy, maternal education, older siblings, ETS, child’s gender, season of the study
Iossifova *et al.* (2009) [44]	No statistically significant associations reported.	
Raaschou-Nielsen *et al.* (2010) [49]	No statistically significant associations reported.	
Choi *et al.* (2010) [53]	**PGE exposure in indoor air** (μg/m^3^) ***One ln-unit (continuous scale)*** aOR 1.5 95%CI (1.0–2.3)	Gender, second hand smoke exposure, allergic diseases in both parents, chemical based cleaning, home construction period, limonene, ln-transformed cat and dog allergen concentrations, BBzP and DEHP concentration in the dust sample
Rosenbaum *et al.* (2010) [50]	**Any wheezing during the first of year *Penicillium Not detected vs. 16-119 vs. 120–1270 (CFU/m^3^)*** Referent *vs.* OR 1.80 95%CI (0.50–6.55) *vs.* OR 6.18 95%CI (1.34–28.46)	All models adjusted for season of visit, maternal smoking during pregnancy, any smoker in home, day care center or non-relative care, endotoxin levels. Total fungi also adjusted for insurance, mother’s education, baby’s race and any living room carpet. Aspergillus also adjusted for insurance, mother’s education and baby’s gender. Penicillium also adjusted for insurance, baby’s gender, mother’s age and baby’s age at mold collection visit. Cladosporium also adjusted for mother’s education, baby’s gender, mother’s age and baby’s age at mold collection visit
Hunt *et al.* (2011) [42]	**Wheezing; *PM_2.5_ (μg/m^3^) < 15 vs.*** ***≥15*** Referent *vs.* aOR 4.21 95%CI (1.36–13.03)	Maternal age and education, gender, season of home visit, living room carpeting
Jones *et al.* (2011) [55]	**Asthma case definition; 85th percentile of indoor air mold levels *Aspergillus (CFU/m^3^)*** Referent *vs.* OR 6.11 95%CI (1.37–27.19)	Age, family history of asthma
Karvonen *et al.* (2012) [47]	**Asthma; *Living room floor Endotoxin (EU/m^2^)*** OR 0.71 95%CI (0.51–0.99) ***Mother’s mattress Amount of dust (mg/m^2^)*** OR 0.73 95%CI (0.58–0.93) **Any wheezing; *Living room floor EPS (EPSU/m^2^)*** OR 0.82 95%CI (0.68–0.99) * **All OR calculated for an interquartile change**	Study centre, farming status, gender, maternal history of allergic diseases (hay fever, atopic dermatitis and/or asthma), smoking during pregnancy and the number of siblings
Moniruzzaman *et al.* (2012) [56]	**Asthma; Child’s bedroom *3.614 vs. 5.324 vs. 9.628 vs. 26.342 (EU/g dust)*** Referent *vs.* aOR 1.44 *p*-value (0.294) *vs.* aOR1.45 *p*-value (0.283) *vs.* aOR2.31 *p*-value (0.016).	Smoking, cleaning habits, crowdedness, family asthma, allergy history
Jedrychowski *et al.* (2014) [45]	**Occurrence of wheezing; *PAH (ln*)** IRR 1.08 95%CI (1.02–1.14) **Recurrent wheezing; *PAH (ln)*** OR 1.61 95%CI (1.16–2.24)	

Abbreviations: IAP, indoor air pollution; aOR, adjusted odds ratio; OR, odds ratio; RR, risk ratio; CI, confidence interval; GM, geometric mean; ln, logarithm natural.

As previously stated, studies that investigated PM exposure also provided inconclusive findings. Tavernier *et al.* reported similar concentrations of PM in the living rooms of cases compared to controls (OR 1.18 95%CI 0.80–1.74) [59] followed by Raaschou-Nielsen *et al.* (2010) who did not confirm the hypothesis of a systematic association between risk for wheezing symptoms and indoor exposure to PM_2.5_ [49]. However, a later study by Hunt *et al.* which quantified PM concentrations in the inner-city homes of a birth cohort whose mothers had a diagnosis of asthma, demonstrated that elevated levels of indoor PM_2.5_ (≥15 μg/m^3^) were a significant risk factor for infant wheezing (OR 4.21 95%CI 1.36–13.03) [42]. Future studies are needed to shed light on the indoor air chemicals’ exposure and children’s respiratory health.

A summary of the exposure categories and significantly associated measures of effect for each health outcome of all included studies is presented in Table 3.

## 4. Discussion

In this review we aimed to summarize existing epidemiological evidence of the association between quantitative estimates of indoor environmental pollutants including both chemical emissions and bioaerosols with respiratory symptoms during infancy and early childhood including wheezing and asthma. Our findings indicate that the most analysed bioaerosols included bacteria-fungi exposure indicators (mainly endotoxin) and dust mites. Evidence is sufficient for endotoxin exposure and asthma/wheezing development and suggestive for other bacteria-fungi indicators including EPS-Pen/Asp and (1->3)-β-D-glucans which is in contrast to the IOM report which characterized evidence of an association of these pollutants with children’s respiratory disease as insufficient [34]. In line with the IOM report, we found that evidence of exposure to pet allergens and adverse respiratory health effects in early ages is limited and that reported findings are contradictory [34]. On the other hand, our review did not confirm a causal relationship of dust mites exposure and asthma development. Finally, the most analysed pollutants among the indoor chemicals considered were VOCs, NO_2_, PM and formaldehyde but evidence of association for all of these was classified as insufficient which is in line with the IOM report [34]. Furthermore, limitations of studies published over the last 10 years remain very similar to those of studies published before 2000 [34]. Many of the studies undertaken are still not based on rigorous protocols and definition of outcome, measurement of exposure, appropriate population selection, and generalizability of the findings are often not adequately addressed. Below we present a critical discussion of the methodological limitations of the studies we considered along with recommendations for future research.

One particular limitation identified is the difficulty of comparing exposure levels and effect associations between studies when different sampling strategies, sampling techniques, and analyses are applied for the pollutants of interest. Endotoxin exposure was primarily assessed through dust samples, however, comparing the endotoxin dust levels between studies is difficult since different dust collection methods and analytical procedures for measuring endotoxin levels are used. Specifically, Horick *et al.* collected airborne samples for endotoxin measurements and reported that the analysis of airborne endotoxin has an impact on the respiratory health of children [41]. These findings support the hypothesis that inhalation is the relevant route of exposure and suggest that although surrogate exposure measures (dust endotoxin) are effective means to identify a role of endotoxin in childhood respiratory disease the magnitude of endotoxin’s effect may be underestimated [41]. In addition, a number of previous studies have shown that the study populations from different countries differ in an number of factors such as household materials and cleaning habits both of which have been identified as predictors for endotoxin levels [60,61,62]. Such variations need to be taken into account for the design of future studies.

The process of dust collection also plays an important role. As previously described, the reservoir dust should be sieved before analysing endotoxin as if it is not sieved the collected dust particles are subjected to a biased exposure estimate. Also different locations in the home and sampling times may yield different endotoxin levels [63]. Specifically, Monizzuramman *et al.* found significant differences of endotoxin concentrations between samples taken from the child’s bedroom *vs.* living room. This is likely due to the living room being the most commonly used place by all family members [56]. Furthermore, higher endotoxin levels in floor dust has been found in homes with indoor pet-keeping [64], in home with agricultural activities [54] and in rural areas [65] and strong predictors for lower levels of endotoxin are vacuum cleaning and ventilation habits [66]. Finally, another important factor to consider in future studies is the seasonal variation of ventilation as endotoxins often come from outdoor sources [56].

Similarly, a potential explanation for the opposite findings for fungal exposure and childhood respiratory disease might be the difference in the exposure assessment approach used. Specifically, EPS-Pen/Asp exposure was mainly measured from house dust while viable fungi were measured from airborne samples. These differences likely arise as they do in endotoxin measurements due to different sampling locations being utilized (urban, suburban or rural areas). We also need to consider that both measurements of endotoxin and EPS are only crude markers of the total microbial exposure and that in different environments may reflect quite different microbial exposures [64,67,68]. Furthermore, indoor microbial measurements of pollutants were performed either from the child’s or mother’s mattresses. However, previous studies have shown the mattresses of infants are often new and the levels of microbes low [67]. Although mother’s mattress dust is only an indirect indicator of child’s exposure, at least for the children who do not sleep with their mothers it reflects the average exposure at home. Since it can be argued that measurement of allergen levels in one house does not accurately reflect the total allergen exposure of the child and therefore a vacuum cleaner dust sample presumably is a better representation of the whole house as it probably reflects the overall microbial status of the home [54]. For this reason, more recent studies have considered combined house dust mean index exposure based on measurements of both house dust allergens taken from the different house sites [46].

Similar limitations were identified for studies that assessed indoor exposure to known chemical hazards. Exposure assessment relied on single point measurements for specific pollutants for the majority of the studies. The primary room of interest among studies was the living room and the sampling duration was relatively short varying between 5 days and 10 weeks during the first months of life. In addition, the majority of the studies used passive samplers for the exposure of interest, which have been found to underestimate the levels of weak sorbents such as benzene [69]. Furthermore, previous studies failed to assess seasonal trends which are known to act as an important factor for IAP levels with higher winter levels for PM and CO_2_ and exhibited higher concentration in warmer seasons for formaldehyde and bioaerosol [70,71]. Moreover, existing studies failed to take into consideration other important factors including ventilation, cleaning condition, cultural habits, outdoor pollution and climate which may alter indoor levels of exposure [71]. As in most studies which have assessed indoor air quality and respiratory symptoms, indoor air sampling was of short duration, performed in specific sites and for specific pollutants and therefore the concentrations do not represent long-term exposure levels, but rather a snapshot of the indoor environment at the time of sampling. Likewise, exposure to these agents cannot be assessed accurately either with questionnaires.

Further to the above, the outcome definition and assessment approaches differ among studies. Studies used either validated questionnaires or doctor diagnosis or maternal diaries or combined information to assess asthma and wheezing. Moreover, there is a potential problem with the validity of the diagnosis of asthma because of the young age of the children recruited and the difficulty in differentiating wheezing illness in this age group. Questionnaire responses are also subject to recall or observational biases, as even with the use of a standardized questionnaire, parents may apply different interpretations of questions in relation to their children’s symptoms. Furthermore, even if we restricted our search to studies with children’s population up to the age of 5, some studies included children of older ages in their total population but due to the small number of studies we decided to include them in this review. In addition, study populations often differed with some studies examining the exposure-effect associations among children at risk of a specific diseases (parental history of atopy/disease or sibling with atopy/disease) and therefore these results should also be interpreted with caution.

In summary, previous studies have used a variety of methodologies to assess indoor exposure to environmental hazards but all of this research has a number of methodological limitations. It is clear that the majority of previous studies failed to assess accurately whole exposures and to capture spatial and temporal variations in exposure as no repeated measurements were performed. In addition, previous studies failed to count for the role of child and family related activities, which are closely, related to later childhood diseases. Despite the existing limitation regarding the quantity and the quality of existing research data more comprehensive epidemiological and interventional studies are needed to resolve many important issues concerning the health effects of indoor air. Further studies are required to improve understanding and quantify the magnitude of individual exposure to air pollution in different types of indoors microenvironments including childcare settings. Future work should take into account different sources of indoor pollution, seasonal variations, home-related activities, lifestyle scenarios, outdoor exposure and mobility of the children. Such an approach will prevent any potential misclassification that could arise when exposure is based solely on specific on site measurements at home. For this reason, the adaptation of innovative techniques for exposure assessment that combine direct all-day personal exposure measurements, direct measurements of micro-environmental concentrations and personal activity information is emerging [72,73].

## 5. Conclusions

In summary, even though the number of current studies is insufficient to provide a definitive conclusion, this review provides a useful summary of existing quantitative research findings. However, due to the small number of studies found, the diversity of the pollutants discussed, and the heterogenous methodology used, it is not possible to determine if the evidence linking these pollutants to respiratory problems during early childhood is conclusive. The results of retrieved studies confirm the shortage of knowledge in this important area and pose the necessity for future well-designed epidemiological cohort studies. Future research must more fully address the complex nature of the indoor environment in order to provide a sound basis for programming proper risk management and communication interventions by local health protection agencies.
